# Development, validation, and web deployment of a rebleeding risk prediction model for acute non-variceal upper gastrointestinal bleeding in a Chinese population

**DOI:** 10.3389/fmed.2025.1716768

**Published:** 2025-12-11

**Authors:** Yujie Chen, Yingkai Xu, Wei Li, Yu Peng, Song Zhang, Weitian Xu, Qingming Wu

**Affiliations:** 1 School of Medicine, Wuhan University of Science and Technology, Wuhan, Hubei, China; 2 Department of Gastroenterology, General Hospital of Central Theater Command, Wuhan, Hubei, China; 3 Department of Transfusion Medicine, General Hospital of Central Theater Command, Wuhan, Hubei, China

**Keywords:** acute non-variceal upper gastrointestinal bleeding, clinical decision support, prognostic model, rebleeding, risk prediction model

## Abstract

**Background:**

Acute non-variceal upper gastrointestinal bleeding (ANVUGIB) is a common life-threatening emergency. Despite advances in endoscopic hemostasis, the 7-day rebleeding rate remains as high as 15–30%. Existing risk assessment tools show limited performance in Chinese populations, underscoring the need for a high-precision model tailored to local patients.

**Objective:**

To develop, validate, and deploy an individualized model for predicting 7-day rebleeding risk in Chinese patients with ANVUGIB using early clinical information.

**Methods:**

We retrospectively included 818 patients with ANVUGIB treated at the General Hospital of Central Theater Command between January 2020 and December 2023, randomly divided into a training cohort (*n* = 572) and an internal validation cohort (*n* = 246) at a 7:3 ratio. An additional 147 patients admitted between January and August 2024 were used as a temporally independent external validation cohort. Predictor variables were selected using least absolute shrinkage and selection operator (LASSO) regression, followed by multivariable logistic regression modeling. Model performance was evaluated using receiver operating characteristic (ROC) curves, calibration plots, and decision curve analysis (DCA), and generalizability was tested in the external validation cohort.

**Results:**

Five independent predictors were identified: syncope, pulse rate, red cell distribution width, serum albumin, and bowel sounds. The prediction model incorporating these variables achieved areas under the curve (AUCs) of 0.843 (95% CI 0.784–0.903), 0.833 (95% CI 0.742–0.924), and 0.825 (95% CI 0.700–0.950) in the training, internal validation, and external validation cohorts, respectively. Calibration plots and decision curve analysis confirmed good consistency and clinical utility.

**Conclusion:**

We developed and validated a 7-day rebleeding risk prediction model for ANVUGIB in a Chinese emergency department population. The model outperformed existing scoring systems, and deployment as a Shiny-based web tool enables early risk identification and individualized decision-making in emergency care.

## 1 Introduction

Acute non-variceal upper gastrointestinal bleeding (ANVUGIB) is one of the most common critical emergencies in clinical practice, with an annual global incidence of approximately 50–160 cases per 100,000 population (1, 2). In recent years, advances in endoscopic hemostasis have improved the initial success rate of bleeding control in patients with ANVUGIB; however, the 7-day rebleeding rate remains high, at approximately 15–30% (3, 4). Rebleeding increases the need for transfusion and repeat interventions, elevates the risk of infection and multiple organ dysfunction, and is closely associated with higher in-hospital mortality (5–8). Therefore, accurately identifying high-risk patients for rebleeding at an early stage of admission and promptly adjusting treatment strategies is crucial for improving clinical outcomes.

Patients with ANVUGIB present with complex clinical conditions. Significant variations exist in bleeding etiology, hemodynamic status, comorbidity burden, and exposure to antithrombotic agents or NSAIDs, resulting in highly heterogeneous rebleeding risk profiles. Currently, no standardized risk stratification criteria for rebleeding are available in clinical practice (9–11). In the early management of patients with ANVUGIB, physicians often face the dual challenges of limited information and time constraints, making it difficult to achieve reliable risk stratification based solely on clinical experience (12). Therefore, a reliable risk prediction tool is of great clinical significance for accurately identifying high-risk patients and guiding therapeutic strategies.

Currently, the most commonly used risk scoring systems for ANVUGIB in clinical practice are the AIMS65 score and the Glasgow-Blatchford score (GBS). However, these tools were not originally designed for predicting rebleeding. AIMS65 primarily assesses the risk of in-hospital mortality, while GBS was initially developed to determine the need for urgent intervention, and neither was specifically intended for rebleeding risk stratification (2, 13). Moreover, numerous studies have demonstrated that their sensitivity and specificity in distinguishing high- from low-risk patients are limited, and their performance in predicting rebleeding outcomes is suboptimal (14, 15). More importantly, as these tools were largely developed in Western populations, their predictive performance decreases when applied to Chinese patients due to differences in etiological spectrum, antithrombotic drug exposure, *Helicobacter pylori* prevalence, and comorbidity profiles, thereby limiting cross-population generalizability (14, 16).

Given these limitations, this study aimed to develop and validate a rebleeding risk prediction model for ANVUGIB in Chinese patients by leveraging single-center retrospective cohort data and integrating multidimensional clinical information available at early admission.

## 2 Materials and methods

### 2.1 Study design and setting

This single-center retrospective cohort study included eligible patients with acute non-variceal upper gastrointestinal bleeding (ANVUGIB) admitted to the General Hospital of Central Theater Command between January 2020 and August 2024. All cases were screened through the hospital’s electronic medical record system. Data collected included demographic characteristics, clinical symptoms, vital signs, laboratory parameters, and in-hospital outcomes. To ensure data quality, data extraction was performed independently by two investigators (double entry) and cross-checked.

### 2.2 Study population and grouping

#### 2.2.1 Inclusion criteria

1) Age ≥18 years.
2) Confirmed diagnosis of ANVUGIB.
3) Completion of upper endoscopy within 48 h of admission with exclusion of variceal bleeding.

#### 2.2.2 Exclusion criteria

1) Confirmed or suspected variceal bleeding due to esophageal/gastric varices.
2) Concurrent upper gastrointestinal malignancy or history of upper gastrointestinal surgery.
3) Self-discharge after admission or >20% missing key variables.
4) Pregnancy or lactation.

#### 2.2.3 Grouping

A total of 818 patients with ANVUGIB admitted to the emergency department between January 2020 and December 2023 were retrospectively included. Patients were randomly assigned to a training cohort (*n* = 572) and an internal validation cohort (*n* = 246) in a 7:3 ratio. An additional 147 patients admitted between January and August 2024 were used as an independent temporal validation cohort.

### 2.3 Definitions

#### 2.3.1 Primary outcome

The primary outcome was rebleeding within 7 days, defined as any of the following after initial hemostasis (pharmacological, endoscopic, or interventional):

1) Endoscopically confirmed fresh or active bleeding.
2) A hemoglobin drop ≥2 g/dL after exclusion of other sources of blood loss.
3) Recurrent hematemesis or melena with hemodynamic instability (systolic blood pressure <90 mmHg or heart rate >100/min).
4) Requirement for repeat endoscopy, interventional embolization, or surgery.

All aforementioned rebleeding events were restricted to those occurring within 7 days after admission.

#### 2.3.2 Key covariates

*Active bowel sounds*: Assessed in a quiet setting with the patient supine. The stethoscope was placed in the right lower quadrant, then moved counterclockwise across four quadrants, with ≥1 min of auscultation per quadrant. Active bowel sounds were defined as ≥10 sounds/min or the presence of high-pitched, hyperactive, metallic, or “tinkling” sounds. Initial assessment was performed by an attending or senior resident and verified by another physician. Discrepancies were adjudicated by a senior gastroenterologist. Patients with recent use of laxatives, prokinetics, or bowel stimulation within 6 h were excluded from bowel sound analysis.

*Altered mental status*: Defined as Glasgow Coma Scale (GCS) ≤13 on admission.

*Hepatic dysfunction*: Serum ALT or AST >2 × upper limit of normal (ULN) at admission.

*Renal dysfunction*: Serum creatinine >110 μmol/L at admission.

### 2.4 Predictors and data collection timeline

#### 2.4.1 Predictors collected

*Demographics*: Age, sex, smoking, alcohol use.

*Comorbidities*: Hypertension, diabetes, coronary artery disease, hepatic dysfunction, chronic kidney disease.

*Medications*: Anticoagulants, antiplatelets, NSAIDs.

*Clinical symptoms*: Hematemesis, melena, syncope.

*Vital signs*: Systolic blood pressure (SBP), pulse, bowel sounds.

*Laboratory tests*: Hemoglobin, platelet count, albumin, creatinine, INR, PT.

*Risk scores*: GBS, AIMS65.

#### 2.4.2 Timing of data collection

All candidate predictors were collected from admission to the time of the index endoscopy. Information related to the first endoscopic examination was obtained within 48 h after admission. Rebleeding events within 7 days after admission were determined based on detailed review of the inpatient clinical course, including progress notes, repeat endoscopic examinations, and treatment records.

#### 2.4.3 Missing data

Missing data were handled according to the proportion of missingness and the assumption that data were missing at random. For candidate predictors with <5% missingness, single imputation was performed using the median for continuous variables and the mode for categorical variables, given the minimal expected impact on effect estimates and model performance. For variables with ≥5% missingness, multiple imputation by chained equations (MICE) with 10 imputations was applied to better account for uncertainty.

#### 2.4.4 Outliers and standardization

Outliers were checked using boxplots and retained if within clinically reasonable ranges. Continuous variables were standardized using Z-scores before modeling.

### 2.5 Model development

#### 2.5.1 Model type

The outcome was 7-day rebleeding (yes/no). A multivariable logistic regression model was applied.

#### 2.5.2 Variable selection

LASSO regression with 10-fold cross-validation was used to identify candidate predictors. These variables were then entered into multivariable logistic regression to develop the final model.

### 2.6 Model validation

#### 2.6.1 Internal validation

The dataset from January 2020 to December 2023 was randomly split (7:3) into training and internal validation cohorts. Model performance was assessed in the internal validation set using AUC, calibration, and decision curve analysis (DCA).

#### 2.6.2 External validation

Patients admitted between January and August 2024 served as an independent temporal validation cohort. The same evaluation methods were applied to test generalizability.

#### 2.6.3 Comparison with existing scores

In the internal validation cohort, AUCs of AIMS65 and GBS were calculated for comparison. AUC differences were tested using DeLong’s method. DCA was further applied to compare net clinical benefit across models at different risk thresholds.

### 2.7 Model performance evaluation

#### 2.7.1 Discrimination

Discrimination was assessed by ROC curve analysis with AUCs and 95% confidence intervals.

#### 2.7.2 Calibration

Calibration was assessed using calibration-in-the-large and calibration slope. The Hosmer–Lemeshow test and bootstrap-derived calibration plots were used to further verify calibration.

#### 2.7.3 Clinical utility

Clinical utility was evaluated using DCA to quantify net benefit across a range of risk thresholds.

### 2.8 Stratified analysis

To evaluate the potential impact of different initial hemostatic strategies on the performance of the rebleeding risk prediction model, a prespecified stratified analysis was conducted based on early intensive hemostatic intervention. The overall cohort was divided into two groups: (1) patients who did not receive early intensive hemostatic intervention; and (2) patients who received early intensive hemostatic intervention. Early intensive hemostatic intervention was defined as the timely implementation, after the index bleeding event, of at least one active hemostatic modality, including early endoscopic hemostasis, transcatheter arterial embolization, or surgical intervention.

In this stratified analysis, the regression coefficients of the primary model were kept unchanged. Predicted probabilities of 7-day rebleeding were calculated for each patient based on the predictors. ROC curves were then developed, and AUCs with 95% confidence intervals were estimated separately in each subgroup to assess the discriminative performance of the model under different initial treatment strategies.

The analysis code and Shiny deployment scripts are available in a public GitHub repository.^1^

### 2.9 Statistical software

All analyses were performed using FreeStatistics software (V2.2.0). Continuous variables were tested for normality using the Shapiro–Wilk test. Normally distributed variables were reported as mean ± SD and compared with independent-samples *t*-test; non-normally distributed variables were presented as median (IQR) and compared with the Mann–Whitney *U* test. Categorical variables were expressed as counts (%) and compared using the *χ*^2^ test or Fisher’s exact test. A two-sided *p* < 0.05 was considered statistically significant.

### 2.10 Ethics statement

This study was approved by the Ethics Committee of the General Hospital of Central Theater Command [(2025)003-01]. Given its retrospective nature, informed consent was waived. All data were anonymized and handled in strict compliance with the Declaration of Helsinki.

## 3 Results

### 3.1 Study population and baseline characteristics

A total of 965 eligible patients with ANVUGIB were included; 88 (9.1%) experienced rebleeding within 7 days. Patients were stratified into a non-rebleeding group (*n* = 877) and a rebleeding group (*n* = 88). Table 1 summarizes the clinical characteristics. Compared with the non-rebleeding group, the rebleeding group showed:

**Table 1:** Baseline characteristics of patients with/without 7-day rebleeding.

Variables	Total (*n* = 965)	Non-rebleeding group (*n* = 877)	Rebleeding group (*n* = 88)	*p*-value
Demographics				
Male, *n* (%)	746 (77.3)	669 (76.3)	77 (87.5)	0.017
Age, (years), mean ± SD	57.6 ± 18.9	56.9 ± 19.0	64.3 ± 17.0	<0.001
Clinical presentation				
Hematemesis, *n* (%)	311 (32.2)	281 (32)	30 (34.1)	0.695
Melena, *n* (%)	792 (82.1)	721 (82.2)	71 (80.7)	0.721
Syncope, *n* (%)	37 (3.8)	29 (3.3)	8 (9.1)	0.015
Medical history				
Peptic ulcer history, *n* (%)	212 (22.0)	190 (21.7)	22 (25)	0.471
Prior GI bleeding, *n* (%)	181 (18.8)	156 (17.8)	25 (28.4)	0.015
NSAID use, *n* (%)	243 (25.2)	218 (24.9)	25 (28.4)	0.464
Antiplatelet use, *n* (%)	178 (18.4)	153 (17.4)	25 (28.4)	0.011
Anticoagulant use, *n* (%)	44 (4.6)	41 (4.7)	3 (3.4)	0.79
Hypertension, *n* (%)	462 (47.9)	416 (47.4)	46 (52.3)	0.386
Diabetes mellitus, *n* (%)	195 (20.2)	179 (20.4)	16 (18.2)	0.62
Ischemic heart disease, *n* (%)	194 (20.1)	177 (20.2)	17 (19.3)	0.847
Smoking, *n* (%)	555 (57.5)	508 (57.9)	47 (53.4)	0.414
Alcohol use, *n* (%)	538 (55.8)	498 (56.8)	40 (45.5)	0.041
Clinical signs				
Altered mental status, *n* (%)	166 (17.2)	136 (15.5)	30 (34.1)	<0.001
Active bowel sounds, *n* (%)	305 (31.6)	257 (29.3)	48 (54.5)	<0.001
Hepatic dysfunction, *n* (%)	28 (2.9)	21 (2.4)	7 (8)	0.01
Chronic kidney disease, *n* (%)	61 (6.3)	50 (5.7)	11 (12.5)	0.012
Pulse rate (/min), median (IQR)	81.0 (73.0, 91.0)	80.0 (73.0, 90.0)	84.5 (74.0, 95.0)	0.049
Laboratory findings				
SBP (mmHg), median (IQR)	123.0 (111.0, 136.0)	124.0 (112.0, 136.0)	114.5 (103.0, 133.8)	0.006
WBC (×10^9^/L), median (IQR)	7.9 (5.9, 10.4)	7.9 (5.9, 10.4)	8.5 (6.3, 11.2)	0.246
NEUT (×10^9^/L), median (IQR)	5.7 (3.9, 7.9)	5.7 (3.8, 7.9)	6.6 (4.4, 8.4)	0.077
RBC (×10^12^/L), median (IQR)	3.3 (2.6, 4.0)	3.3 (2.6, 4.0)	3.0 (2.3, 3.6)	0.004
HB (g/L), median (IQR)	95.0 (72.0, 117.0)	96.0 (74.0, 118.0)	88.0 (68.0, 102.2)	0.001
HCT (%), median (IQR)	29.3 (22.8, 35.7)	29.5 (23.0, 35.9)	28.0 (21.3, 32.0)	0.01
RDW (%), median (IQR)	13.5 (12.8, 14.9)	13.4 (12.7, 14.7)	14.8 (13.6, 16.9)	<0.001
PLT (×10^9^/L), median (IQR)	209.0 (172.0, 259.0)	209.0 (173.0, 259.0)	201.5 (154.8, 256.2)	0.456
PT(s), Median (IQR)	12.0 (11.3, 12.7)	11.9 (11.3, 12.7)	12.3 (11.6, 12.8)	0.007
INR, median (IQR)	1.1 (1.0, 1.2)	1.1 (1.0, 1.2)	1.1 (1.1, 1.2)	0.004
FIB (g/L), median (IQR)	3.4 (2.9, 3.9)	3.4 (2.9, 3.9)	3.5 (2.9, 4.2)	0.695
D-dimer (μg/mL), median (IQR)	0.1 (0.1, 0.3)	0.1 (0.1, 0.3)	0.2 (0.1, 0.5)	<0.001
CRP (mg/L), median (IQR)	2.0 (0.5, 6.3)	1.8 (0.5, 5.9)	5.0 (1.0, 12.3)	0.003
ALT (U/L), median (IQR)	16.0 (11.0, 23.0)	16.0 (11.0, 23.0)	15.8 (11.8, 22.0)	0.733
AST (U/L), median (IQR)	20.0 (17.0, 25.0)	20.0 (17.0, 25.0)	19.5 (16.0, 26.0)	0.468
ALB (g/L), median (IQR)	38.5 (34.7, 42.1)	38.9 (35.2, 42.5)	33.2 (29.8, 36.6)	<0.001
BUN (mmol/L), median (IQR)	9.3 (6.3, 13.7)	9.0 (6.1, 13.4)	12.3 (8.3, 16.7)	<0.001
Cr (μmol/L), median (IQR)	72.0 (59.0, 90.0)	71.0 (59.0, 88.0)	80.5 (65.8, 103.8)	<0.001
Ca^2+^ (mmol/L), median (IQR)	2.1 (2.1, 2.3)	2.2 (2.1, 2.3)	2.1 (2.0, 2.2)	0.003
K^+^(mmol/L), median (IQR)	4.1 (3.8, 4.4)	4.1 (3.8, 4.4)	4.3 (3.9, 4.7)	<0.001
Risk scores				
GBS, median (IQR)	8.0 (6.0, 11.0)	8.0 (5.0, 11.0)	11.0 (9.0, 12.0)	<0.001
AIMS65, median (IQR)	1.0 (0.0, 2.0)	0.0 (0.0, 1.0)	1.0 (0.0, 2.0)	<0.001

Values are presented as mean ± standard deviation (SD), median (interquartile range, IQR), or number (percentage), as appropriate. Continuous variables were compared using the *t*-test or Mann–Whitney *U* test, and categorical variables were compared using the *χ*^2^ test or Fisher’s exact test.

*Demographics*: Older age (64.3 ± 17.0 vs. 56.9 ± 19.0 years, *p* < 0.001) and a higher proportion of males (87.5% vs. 76.3%, *p* = 0.017).

*Clinical presentation*: Syncope was more frequent (9.1% vs. 3.3%, *p* = 0.015); rates of hematemesis were similar (34.1% vs. 32.0%, *p* = 0.695).

*Comorbidities*: Higher prevalence of hepatic dysfunction (8.0% vs. 2.4%, *p* = 0.010) and chronic kidney disease (12.5% vs. 5.7%, *p* = 0.012).

*Vital signs*: Lower admission SBP (114.5 vs. 124 mmHg, *p* = 0.006), faster pulse rate (84.5 vs. 80 beats/min, *p* = 0.049), and more frequent active bowel sounds (54.5% vs. 29.3%, *p* < 0.001).

*Laboratory tests*: Lower hemoglobin and hematocrit; higher RDW (14.8% vs. 13.4%, *p* < 0.001); more pronounced coagulopathy (prolonged PT and elevated INR, both *p* < 0.01); lower albumin (33.2 vs. 38.9 g/L, *p* < 0.001); and higher C-reactive protein.

*Risk scores*: Higher GBS (median 11 vs. 8, *p* < 0.001) and AIMS65 (median 1 vs. 0, *p* < 0.001).

These differences indicate a higher baseline risk burden among patients who rebled, particularly with respect to coagulopathy, hypoalbuminemia, abnormal bowel sounds, and circulatory instability.

When baseline characteristics were compared between the 2020–2023 development cohort (*n* = 818) and the 2024 temporal validation cohort (*n* = 147) (Supplementary Table S1), age, sex, haemodynamic status, comorbidities and most laboratory indices were broadly similar. Patients in the temporal validation cohort were more likely to present with haematemesis and altered mental status, had slightly higher rates of NSAID and antiplatelet use, but lower proportions of current smokers and drinkers; C-reactive protein levels were modestly higher and serum calcium levels slightly lower. In contrast, the distributions of the five model predictors (syncope, pulse rate, active bowel sounds, RDW and albumin) overlapped closely between the two cohorts, and the 7-day rebleeding rates were almost identical (9.2% vs. 8.8%). Overall, the temporal validation cohort therefore represents a “somewhat sicker but not fundamentally different” case-mix, without the emergence of completely new phenotypes compared with the development data, and the model maintained good performance under these less tightly controlled, more real-world conditions.

### 3.2 Variable selection and prediction model

**Figure 1:**
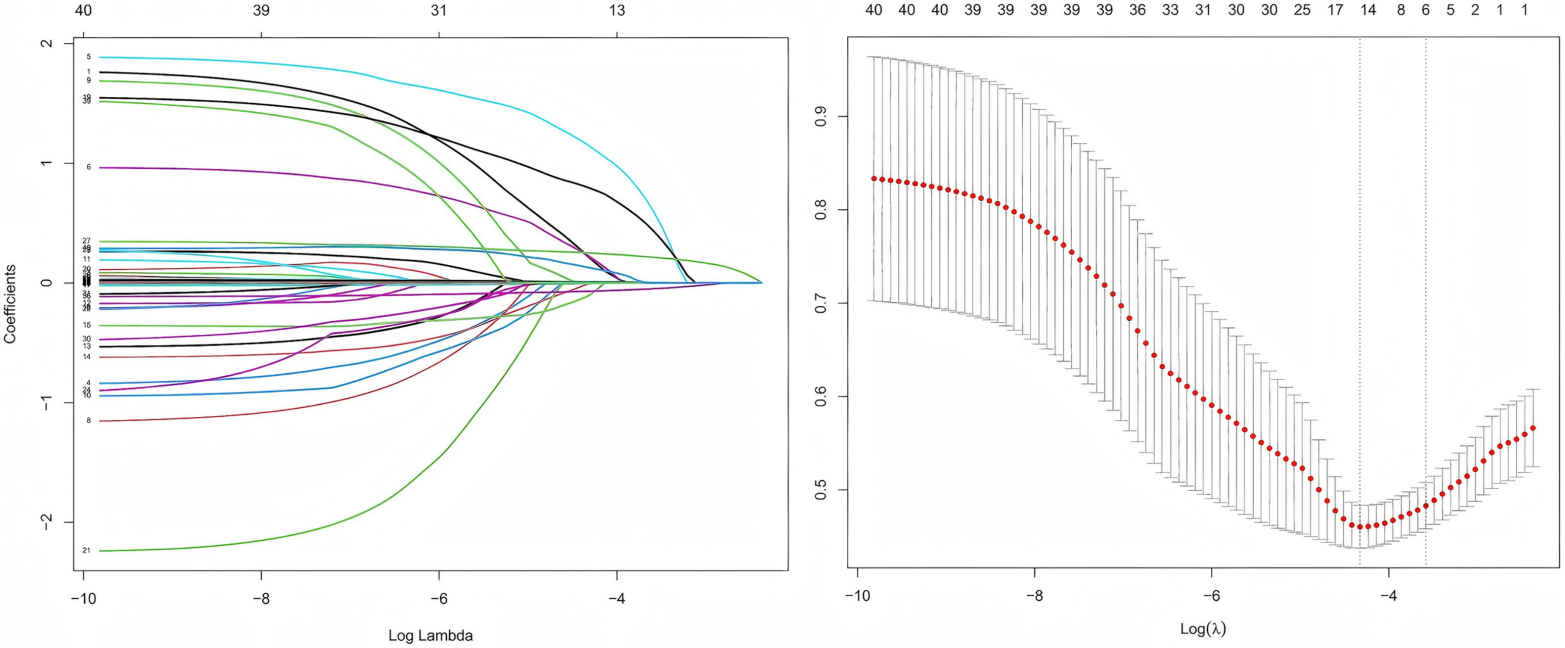
LASSO regression with cross-validation. Coefficient profiles of candidate variables across log(*λ*) values (left). Ten-fold cross-validation was applied to identify the optimal *λ* (right). The *λ* with minimum error and the 1-SE criterion are indicated by dotted lines. Variables with non-zero coefficients at the optimal *λ* were retained for multivariable analysis.

**Figure 2:**
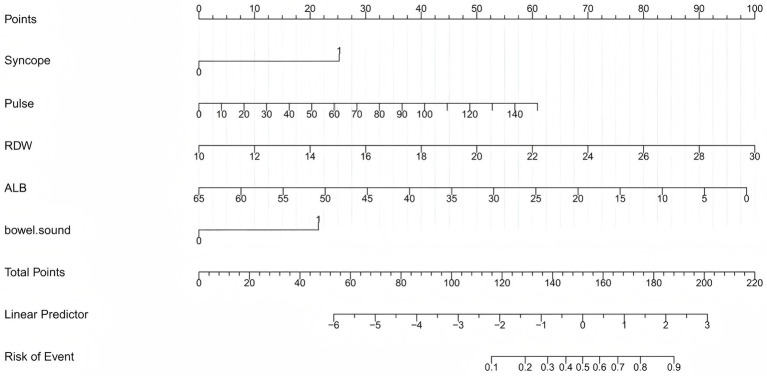
Nomogram for predicting 7-day rebleeding risk in patients with ANVUGIB. The nomogram was developed using five independent predictors: syncope, pulse rate, red cell distribution width (RDW), albumin (ALB), and active bowel sounds. For each patient, the score for each variable is located on the corresponding axis, and the total score corresponds to the predicted probability of 7-day rebleeding.

**Figure 3:**
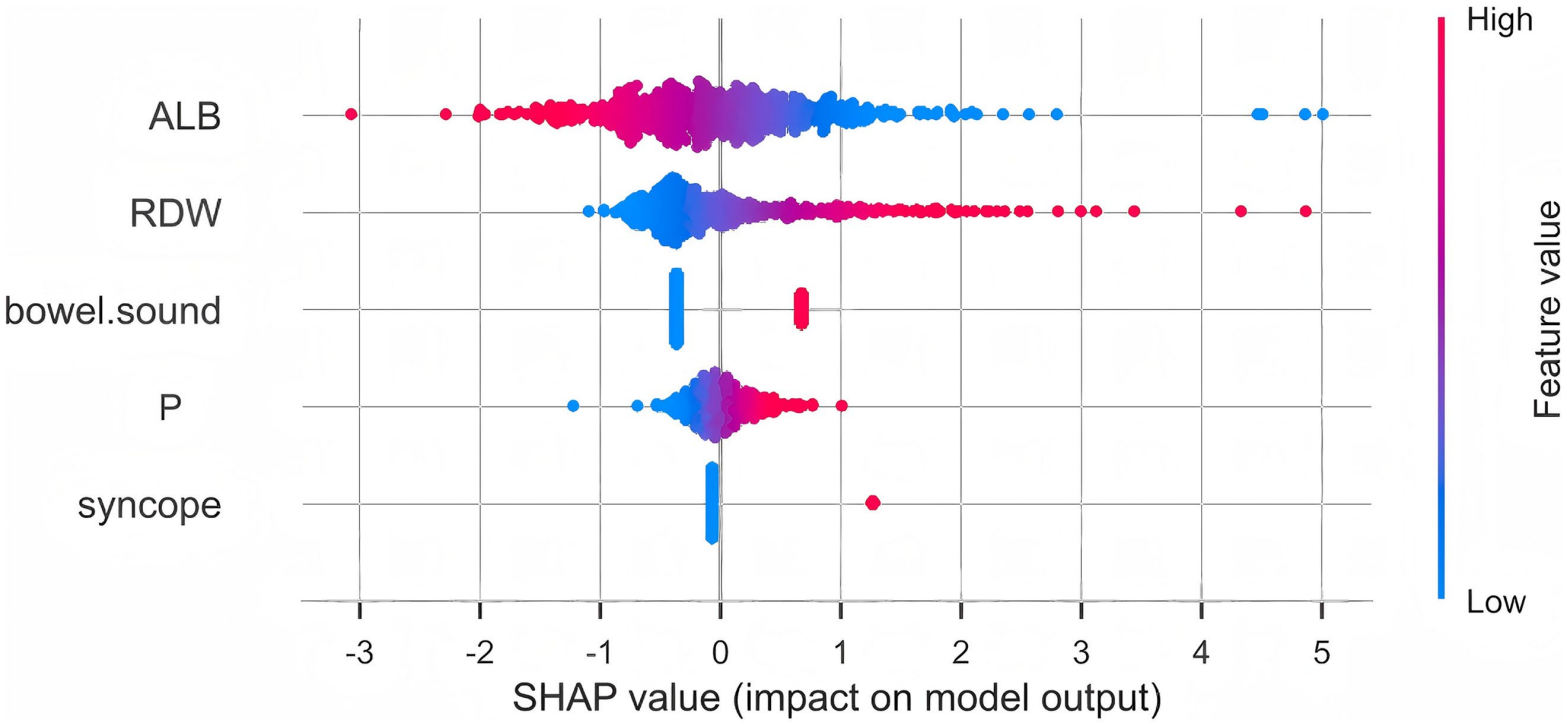
SHAP summary plot of the prediction model. SHAP values indicate the contribution and direction of each variable to the prediction of 7-day rebleeding risk. Lower albumin, higher red cell distribution width (RDW), active bowel sounds, higher pulse rate, and syncope were associated with increased predicted risk. The color gradient represents feature values (red = high, blue = low).

Least absolute shrinkage and selection operator (LASSO) regression with 10-fold cross-validation (Figure 1) identified six variables with non-zero coefficients: syncope, pulse rate, RDW, albumin (ALB), active bowel sounds, and PT. Multivariable logistic regression (Table 2) retained five independent predictors of 7-day rebleeding: syncope (OR = 4.648, 95% CI 1.506–14.627), pulse rate (OR = 1.025, 95% CI 1.003–1.047), RDW (OR = 1.352, 95% CI 1.203–1.519), ALB (OR = 0.914, 95% CI 0.875–0.954), and active bowel sounds (OR = 3.561, 95% CI 1.807–7.016). A nomogram based on these predictors (Figure 2) enables bedside individualized risk estimation. To enhance interpretability, a SHapley Additive exPlanations (SHAP) summary plot (Figure 3) demonstrated the directions and magnitudes of effects: lower ALB, higher RDW, active bowel sounds, tachycardia, and syncope were associated with increased rebleeding risk.

**Table 2:** Multivariable logistic regression of 7-day rebleeding risk in ANVUGIB.

Variable	OR_95% CI	*p*_value
Syncope	4.648 (1.506–14.627)	0.008
Pulse rate	1.025 (1.003–1.047)	0.025
RDW	1.352 (1.203–1.519)	<0.001
ALB	0.914 (0.875–0.954)	<0.001
Bowel sounds	3.561 (1.807–7.016)	<0.001
PT	1.008 (0.975–1.043)	0.625

Odds ratios (ORs) with 95% confidence intervals (CIs) are presented. Variables with *p* < 0.05 were considered statistically significant independent predictors.

### 3.3 Discrimination

**Figure 4:**
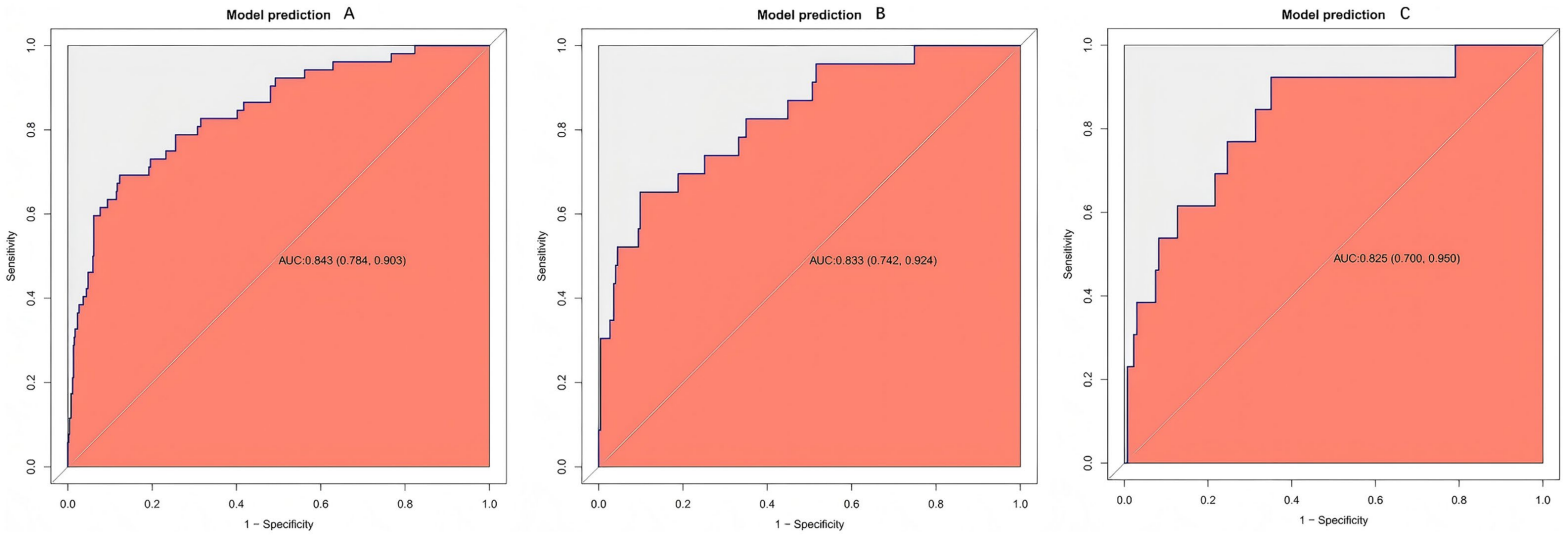
Receiver operating characteristic (ROC) curves of the prediction model. ROC curves are shown for the training **(A)**, internal validation **(B)**, and external validation **(C)** cohorts. The model consistently demonstrates good discrimination, as indicated by curves that are well above the diagonal reference line, reflecting a strong ability to distinguish between patients with and without rebleeding across all datasets.

The model yielded AUCs of 0.843 (95% CI 0.784–0.903) in the training cohort, 0.833 (95% CI 0.742–0.924) in the internal validation cohort, and 0.825 (95% CI 0.700–0.950) in the external validation cohort (Figures 4A–C), indicating stable performance and good generalizability across datasets.

### 3.4 Calibration

**Figure 5:**
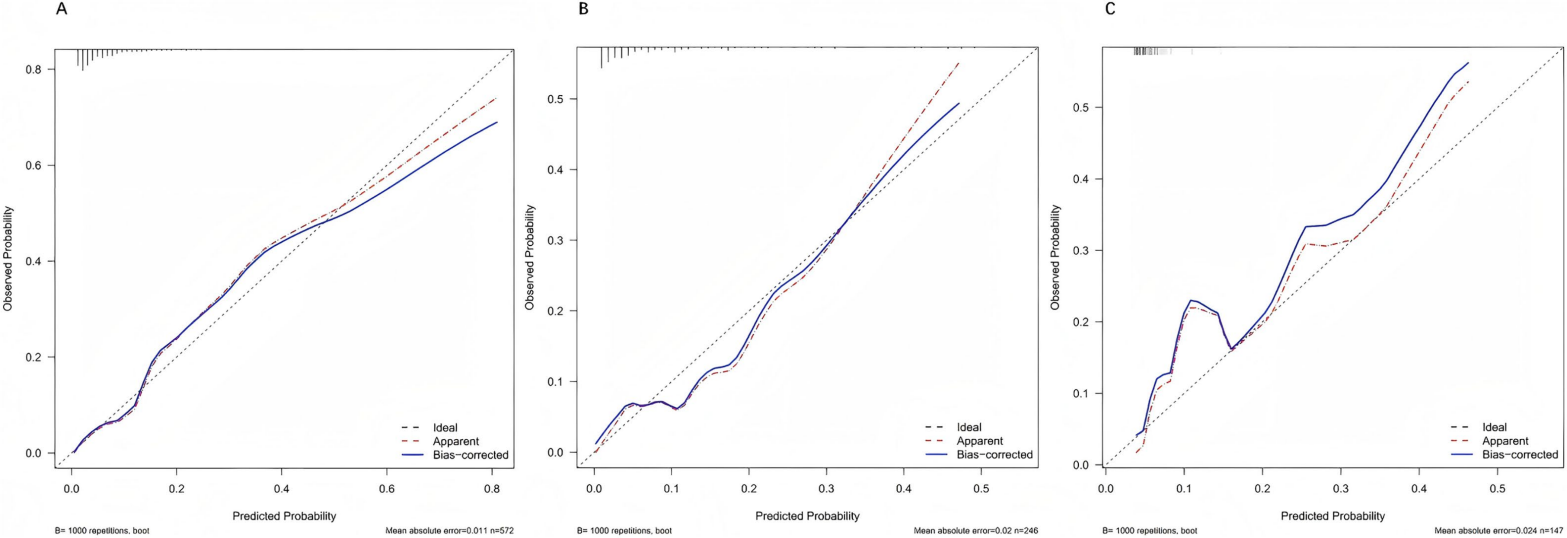
Calibration curves of the prediction model. Calibration plots are presented for the training **(A)**, internal validation **(B)**, and external validation **(C)** cohorts. In all datasets, the bias-corrected curves (blue) closely align with the ideal 45° line (dashed), indicating good agreement between predicted and observed probabilities. This suggests that the model provides reliable risk estimates without systematic over- or under-prediction.

After 1,000 bootstrap resamples, the apparent and optimism-corrected calibration curves showed good agreement with the ideal line in the training, internal validation, and external validation cohorts (Figures 5A–C), indicating satisfactory concordance between predicted probabilities and observed risks (*p* > 0.05).

### 3.5 Clinical net benefit (decision curve analysis)

**Figure 6:**
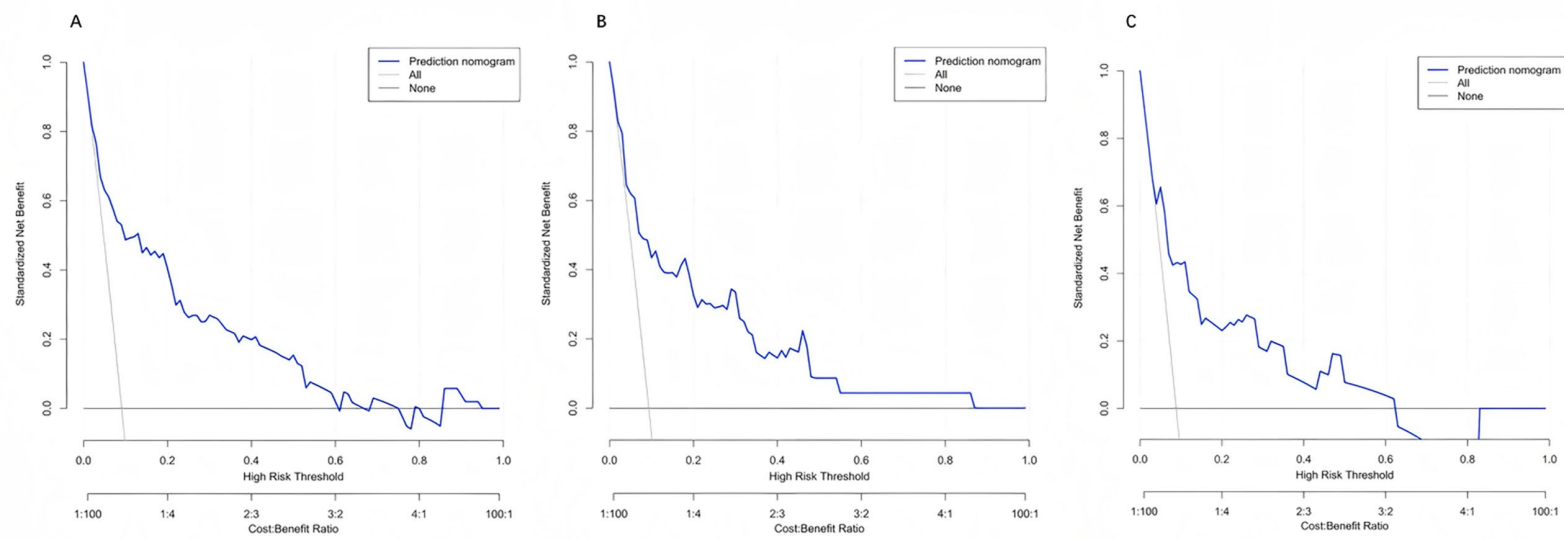
Decision curve analysis (DCA) of the prediction model. Decision curves for the training **(A)**, internal validation **(B)**, and external validation **(C)** cohorts are shown. Across a wide range of threshold probabilities, the model (blue line) yields a higher net benefit compared with the “treat-all” (gray line) and “treat-none” (black line) strategies, indicating superior clinical utility for guiding individualized decision-making.

Across clinically relevant threshold probabilities of 1–60%, the model provided the highest net benefit and consistently outperformed the treat-all and treat-none strategies over a broad range (Figures 6A–C).

### 3.6 Comparison with existing scores

**Figure 7:**
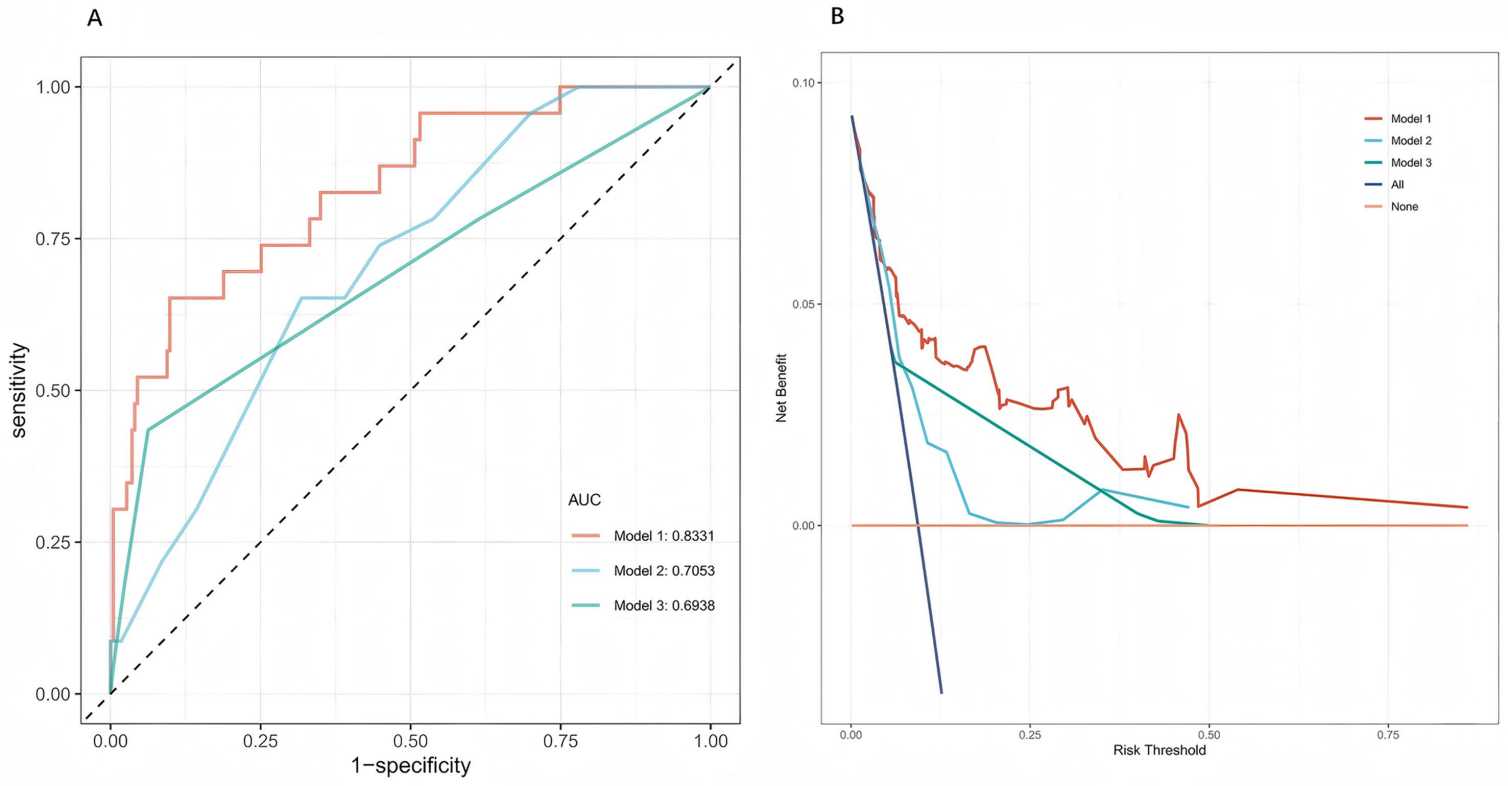
Comparison of model performance in the internal validation cohort. **(A)** Receiver operating characteristic (ROC) curves of the present five-predictor rebleeding risk model (Model 1), the Glasgow-Blatchford score (GBS, Model 2), and the AIMS65 score (Model 3). The new prediction model (Model 1) showed higher discriminative ability than the two existing scores. **(B)** Decision curve analysis (DCA) comparing the clinical net benefit of the three models. Across a wide range of threshold probabilities, the new model provided greater net clinical benefit than GBS and AIMS65.

In the internal validation cohort, the model outperformed conventional scores (Figure 7). ROC analysis showed an AUC of 0.833 for the present model, exceeding that of GBS (AUC = 0.705) and AIMS65 (AUC = 0.694). DeLong’s test confirmed statistically significant differences versus both GBS and AIMS65 (*p* < 0.01). DCA further demonstrated higher net clinical benefit across most thresholds, supporting superior discrimination and clinical applicability in Chinese patients with ANVUGIB.

### 3.7 Stratified analysis of early intensive hemostatic intervention

The model maintained good discriminative performance across all subgroups. In patients who did not receive early intensive hemostatic intervention, the AUC for predicting 7-day rebleeding was 0.816 (95% CI 0.743–0.890). In patients who received early intensive hemostatic intervention, the AUC was 0.862 (95% CI 0.746–0.979). Compared with the overall cohort (AUC 0.843, 95% CI 0.784–0.903), the confidence intervals for subgroup AUCs clearly overlapped with the overall estimate, indicating no substantial attenuation of performance or inconsistent trends (Supplementary Figure S1). These findings suggest that, even under different initial hemostatic strategies, the prediction model developed using clinical information available at admission and before endoscopy maintains stable discriminative ability.

### 3.8 Web deployment

To enhance accessibility and clinical usability, the model was deployed as an interactive web-based calculator using the Shiny framework, accessible via desktop and mobile devices.^2^ At present, the online calculator is hosted on the free-tier plan of the shinyapps.io cloud service. Clinicians can input patient variables to obtain immediate 7-day rebleeding probabilities with visual guidance, facilitating rapid bedside decision-making in the emergency department and wards. The tool implements the fixed regression coefficients and predefined data processing pipeline solely for inference (no online training), ensuring consistency between online and offline predictions. It adheres to the principles of “minimal data collection” and “zero persistent storage” and does not retain identifiable information, thereby meeting institutional data security and privacy requirements. A schematic illustration of the Shiny web application deployment is provided in Supplementary Figure S2.

## 4 Discussion

In this study, we developed a 7-day rebleeding risk prediction model for ANVUGIB based on early clinical information from 965 patients. Using LASSO regression combined with multivariable logistic regression and validating the model with a temporally split external cohort, we demonstrated consistently strong predictive performance in both internal and external datasets. Clinically, rebleeding in ANVUGIB markedly increases mortality risk, while existing scoring systems fall short of early prediction needs. By integrating multidimensional clinical data with multivariable modeling and interpretability analyses, we systematically explored risk factors for rebleeding. We identified low serum albumin, elevated RDW, active bowel sounds, increased pulse rate, and syncope as independent predictors. These findings are directionally consistent with prior evidence (17–25).

Albumin depletion reduces colloid osmotic pressure, leading to mucosal and submucosal edema and microcirculatory impairment. Impaired antioxidant and carrier functions of albumin, along with insufficient synthesis of coagulation factors and extracellular matrix proteins, may delay granulation and epithelial repair, ultimately weakening the clot–tissue interface and increasing rebleeding risk (17–19). In our cohort, serum albumin, originally included in AIMS65 for mortality risk stratification, also demonstrated independent predictive value for rebleeding. Early recognition and supplementation in hypoalbuminemic patients may improve clot stability.

Elevated RDW has been linked to adverse outcomes in multiple cohorts, potentially through inflammation–iron metabolism pathways (IL-6/hepcidin axis) causing iron sequestration and ineffective erythropoiesis. The resulting anisocytosis and impaired deformability reduce oxygen delivery and delay mucosal healing. In addition, systemic inflammation and abnormal red cell morphology may enhance fibrinolysis and destabilize microthrombi, thereby contributing to higher rebleeding risk (20–22). Clinically, patients with elevated RDW warrant attention to inflammation control, assessment of iron metabolism, nutritional support, and optimization of erythropoiesis and oxygen transport.

Active bowel sounds may reflect enhanced intestinal motility and luminal pressure fluctuations triggered by intraluminal hemoglobin breakdown products, disturbing the fragile “soft clot–fibrin mesh” formed after endoscopic hemostasis. This represents a bedside sign of unstable hemostasis or ongoing bleeding (23). As bowel sound activity has rarely been systematically studied, our findings highlight its potential role in risk stratification. Close monitoring and lower thresholds for repeat endoscopy may be appropriate in such patients.

Increased pulse rate generally reflects sympathetic activation and hypovolemia, leading to higher shear stress and “mechanical fatigue” on early hemostatic clots. Although its predictive strength is less pronounced than syncope or bowel sounds, sustained tachycardia may accumulate risk, especially under inadequate resuscitation. Prompt assessment of volume status and optimization of resuscitation are therefore essential (24).

Syncope, in contrast, directly reflects global hypoperfusion and hemodynamic instability due to acute blood loss, indicating a larger initial bleed or unstable hemostasis and strongly signaling high rebleeding risk (25). Such patients should be prioritized for resuscitation and early aggressive intervention.

Collectively, these predictors converge pathophysiologically on rebleeding risk. Our model demonstrated superior discrimination, robust calibration, and greater generalizability compared with conventional scores. In contrast, AIMS65 and GBS both achieved AUCs below 0.71 in our internal validation cohort, consistent with other Asian studies and underscoring their limited generalizability to Chinese populations (14). Beyond statistical performance, clinical deployability defines a model’s true value.

While predictive models have proliferated in recent years, most remain theoretical and lack interactive deployment for clinical use (26). Our web-based Shiny application enables real-time access on desktop and mobile devices, overcoming the “good-on-paper but impractical” limitation of many models. Moreover, the integration of SHAP-based interpretability allows visualization of individual variable contributions, increasing clinician trust and facilitating more precise interventions.

The current web deployment of the model relies on a cloud-based free hosting service (shinyapps.io free tier), which introduces uncertainty in server uptime, response speed, and long-term availability. The application may enter a sleep mode after periods of inactivity or become temporarily slow or unavailable when usage limits are reached or shared resources are constrained. Therefore, the current online implementation should be regarded as a convenient research and demonstration tool rather than a production-grade clinical information system. To date, the model has only undergone pilot implementation within our hospital and remains intended for research and educational use rather than routine clinical decision-making. We are currently advancing its technical integration with the electronic health record (EHR) system. After completion of multicenter validation and information security, privacy, and regulatory compliance review, we plan to migrate the model to an institution-supported paid hosting environment and embed it into EHR workflows via in-house APIs or interfaces based on FHIR/HL7 standards, with continuous maintenance and monitoring by the hospital information technology team. For patients with suspected or confirmed ANVUGIB, the system will then automatically generate individualized 7-day rebleeding risk estimates and risk stratification prompts at the time of data entry, thereby providing stable and sustainable automated decision support for early intervention in routine clinical care.

From a methodological and implementation perspective, the main contributions of this study can be summarised as follows:

- We developed a simple, five-predictor model based entirely on information available before endoscopy in the emergency department, directly targeting 7-day rebleeding rather than mortality or the need for intervention.
- We evaluated the model using a temporally distinct cohort from the same hospital, providing a pragmatic test of performance under moderate temporal changes in case-mix and practice.
- We combined a bedside nomogram, a publicly accessible Shiny web calculator and SHAP-based visualisation of individual predictor contributions to enhance clinical usability and interpretability.
- We demonstrated that, in a Chinese ANVUGIB population, the model outperformed commonly used scores (GBS and AIMS65) in terms of discrimination and net benefit, and provided a transparent, reproducible pipeline with a deployed tool and accompanying documentation.

### 4.1 Limitations and deployment-related risks

This study has several limitations. First, it was a single-centre retrospective study with a limited sample size, and selection bias cannot be fully excluded. Second, although we performed temporal external validation within the same institution, multicentre prospective validation is still lacking, and the model’s performance in other hospitals and healthcare systems remains uncertain. Third, assessment of bowel sounds relied on routine clinical examination and is subject to inter-observer variability; more standardised training and objective assessment tools are needed in future studies.

In addition, in line with recent guidelines on the development and deployment of machine learning tools in healthcare (27–30), several risks and constraints related to model generalisability and the deployed web application should be explicitly acknowledged. The model was deliberately restricted to five easily obtainable predictors to preserve bedside usability. This parsimony improves interpretability and feasibility in busy emergency settings but, as with any model developed in a single cohort, performance may be affected if case mix, disease epidemiology or management pathways differ substantially from those in the development setting. In this study, discrimination and calibration remained stable in the temporal cohort despite these modest shifts, but more pronounced temporal or geographic changes could still cause dataset shift and model drift. Accordingly, use in new environments should be accompanied by context-specific checks, local validation where feasible, and ongoing performance monitoring, with recalibration or updating considered if evidence of drift emerges. The predicted probabilities are designed to support, rather than replace, clinical judgement, and applying the tool outside the intended target population or relying on it as the sole basis for high-stakes decisions would not be appropriate.

From a technical perspective, the current web implementation uses the shinyapps.io free-tier cloud platform, which is suitable for research-oriented prototypes but does not provide the same guarantees of uptime, latency, redundancy and governance as a clinical-grade information system. Occasional latency, automatic “sleep mode,” temporary unavailability and dependence on local internet connectivity may therefore occur. In line with current guidelines on AI deployment in healthcare, the present implementation should be viewed as a convenient research and education tool, while any broader or routine clinical use would require migration to institutionally supported infrastructure, formal integration within hospital IT workflows and, where applicable, regulatory review. Situations in which particular caution is warranted include use in populations or healthcare systems that differ markedly from the development cohort, major temporal changes in ANVUGIB epidemiology or practice, systematic data quality issues, and extreme or implausible inputs; these considerations highlight the importance of prospective multicentre evaluation and a predefined strategy for monitoring and, if needed, recalibration as the tool is adopted more widely.

## 5 Conclusion

Based on early clinical information from 965 patients with ANVUGIB in the emergency setting of the General Hospital of Central Theater Command, we developed and validated a 7-day rebleeding risk prediction model. The model demonstrated excellent discrimination and stable calibration in both internal and external validation cohorts, while SHAP-based analyses illustrated the contributions of key predictors, including hypoalbuminemia, elevated RDW, active bowel sounds, tachycardia, and syncope.

Compared with conventional scoring systems, the model showed superior local applicability and was successfully deployed as an interactive Shiny-based web tool, ensuring clinical operability. In the future, the model will be seamlessly integrated into the EHR system to provide automated decision support across emergency and inpatient care. It has the potential to facilitate early risk identification, precise risk stratification, and individualized management for patients with ANVUGIB, while laying the groundwork for multicenter prospective validation and broader clinical adoption.

## Data Availability

The raw data supporting the conclusions of this article will be made available by the authors, without undue reservation.

## Footnotes

1
https://github.com/maomaoyu152/anvugib-7d-rebleeding-model/tree/main

2
https://acutenon-varicealuppergastrointestinalbleeding.shinyapps.io/rebleeding-risk-anvugib/

## Supplementary material

SUPPLEMENTARY FIGURE S1 ROC curves stratified by early intensive hemostatic intervention. **(A)** Patients without early intensive hemostatic intervention. **(B)** Patients with early intensive hemostatic intervention.

SUPPLEMENTARY FIGURE S2 Schematic diagram of the Shiny web application deployment.
