# Supplementary figures and images for: Development, validation, and web deployment of a rebleeding risk prediction model for acute non-variceal upper gastrointestinal bleeding in a Chinese population
Source: Front Med (Lausanne). 2025 Dec 11;12:1716768. doi: 10.3389/fmed.2025.1716768 (PMC12738914; doi:10.3389/fmed.2025.1716768)

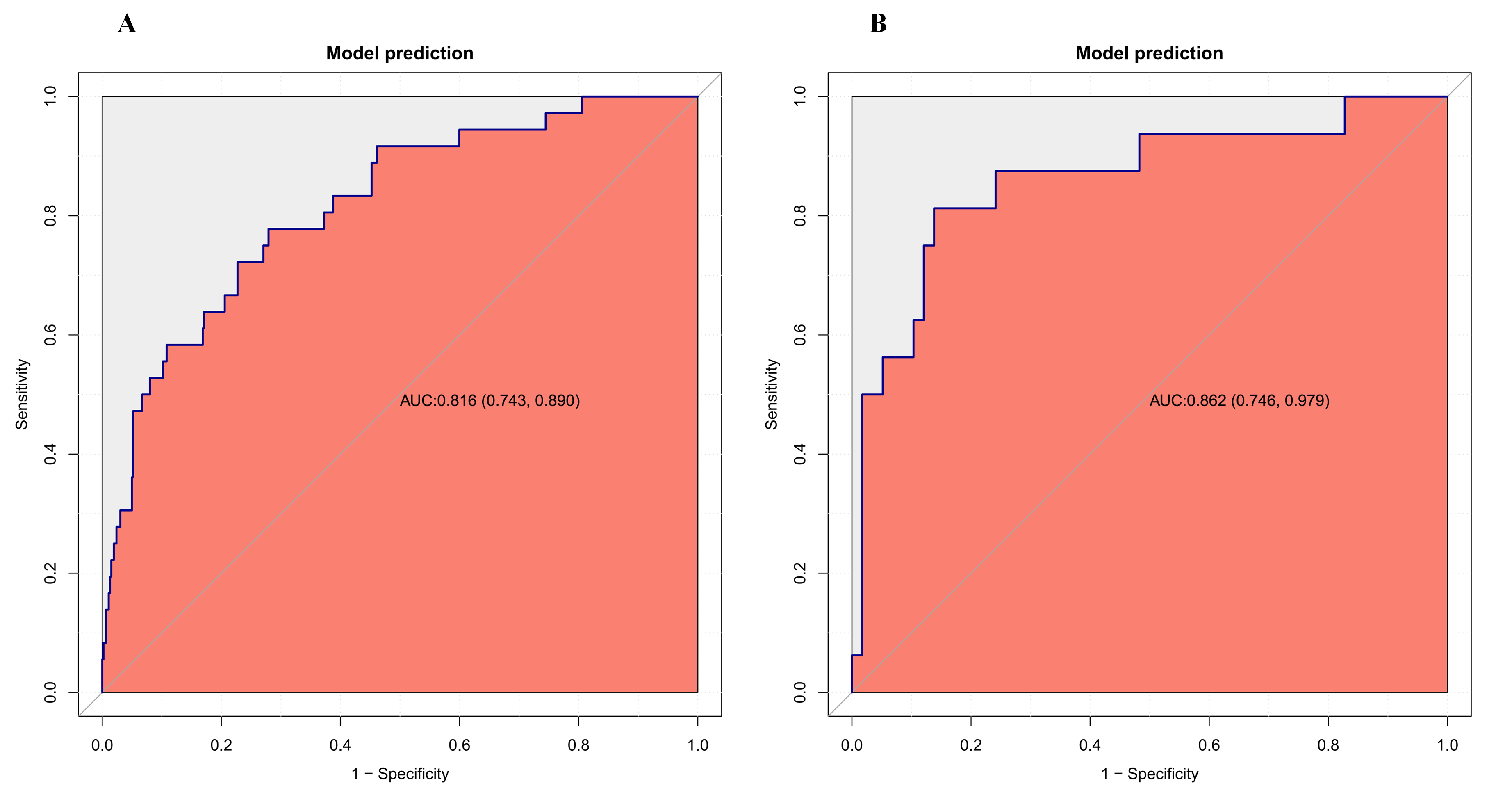

Supplement: SUPPLEMENTARY FIGURE S1 — ROC curves stratified by early intensive hemostatic intervention. (A) Patients without early intensive hemostatic intervention. (B) Patients with early intensive hemostatic intervention. [file Image_1.TIF]

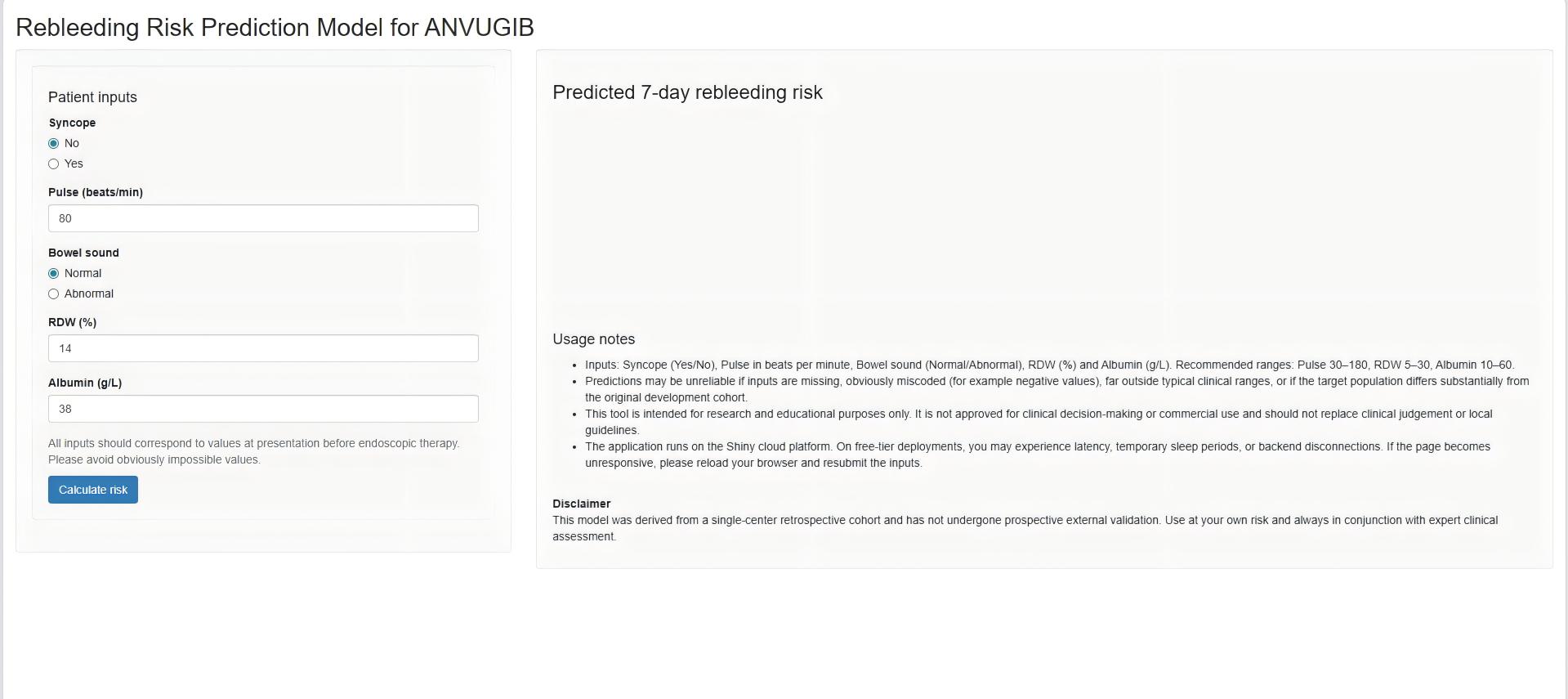

Supplement: SUPPLEMENTARY FIGURE S2 — Schematic diagram of the Shiny web application deployment. [file Image_2.JPEG]

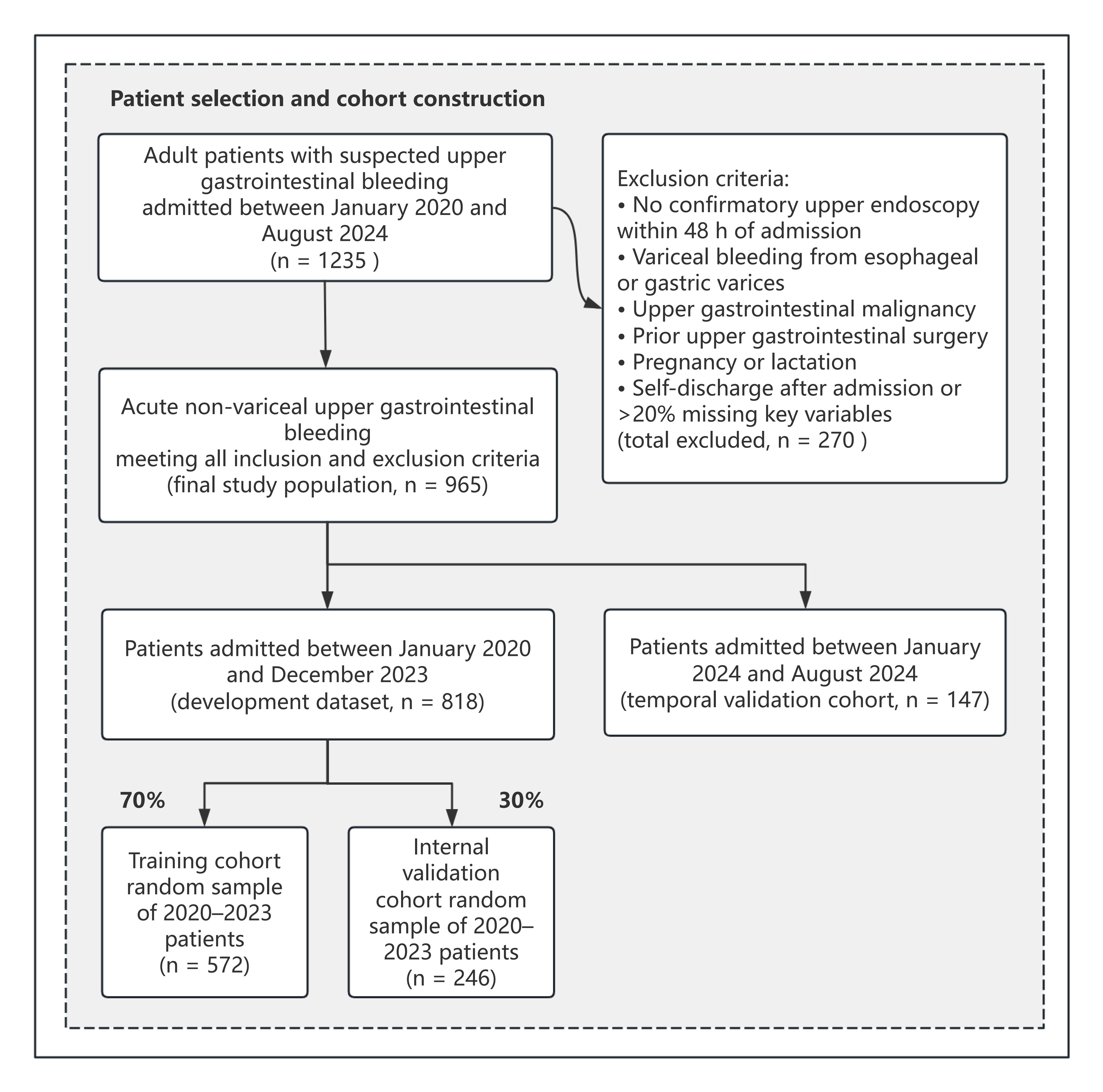

Supplement: Supplementary file 3 [file Image_3.JPEG]
