# Supplementary material for: Development, validation, and web deployment of a rebleeding risk prediction model for acute non-variceal upper gastrointestinal bleeding in a Chinese population
Source: Front Med (Lausanne). 2025 Dec 11;12:1716768. doi: 10.3389/fmed.2025.1716768 (PMC12738914; doi:10.3389/fmed.2025.1716768)
Supplement: Supplementary file 4 [file Data_Sheet_1.DOCX]

**1. Study design and cohort construction**

## 1. Overall study design and data collection workflow

This study was a single-centre retrospective cohort conducted at the General Hospital of Central Theater Command, People’s Liberation Army (PLA), China. Using the hospital’s electronic medical record (EMR) system, we consecutively screened all adult patients with suspected upper gastrointestinal bleeding (UGIB) who presented to our hospital between 1 January 2020 and 31 August 2024.

For all potentially eligible admissions, we extracted the following information:

- Demographic characteristics;

- Presenting symptoms and initial clinical features;

- Vital signs at admission;

- History of comorbid diseases;

- Exposure to relevant medications;

- Laboratory test results;

- Upper endoscopy findings;

- In-hospital outcomes.

Data collection followed a predefined case report form (CRF). Two investigators independently performed data entry (double data entry), followed by cross-checking of all records. Any discrepancies were resolved by discussion between the two investigators, and, when necessary, adjudicated by a senior gastroenterologist.

The primary objective of this study was to develop and validate a multivariable prediction model to estimate the 7-day rebleeding risk in patients with acute non-variceal upper gastrointestinal bleeding (ANVUGIB), using clinical information available early after emergency department (ED) presentation.

- Data from patients admitted between 1 January 2020 and 31 December 2023 were used for model development and internal validation.

- Data from patients admitted between 1 January 2024 and 31 August 2024 were used for temporal external validation.

- The same inclusion and exclusion criteria were applied consistently across both periods.

## 2. Inclusion and exclusion criteria

### 2.1 Inclusion criteria

Patients were included in the study if all of the following criteria were met:

1. Age ≥18 years at admission;

2. Clinical presentation suggestive of upper gastrointestinal bleeding (e.g. haematemesis and/or melena) at the index visit;

3. Diagnosis of acute non-variceal upper gastrointestinal bleeding (ANVUGIB) confirmed by upper endoscopy;

4. Completion of upper endoscopy within 48 hours after admission, with endoscopic exclusion of oesophageal or gastric variceal bleeding.

### 2.2 Exclusion criteria

Patients were excluded if any of the following conditions were present:

1. Failure to complete confirmatory upper endoscopy within 48 hours after admission;

2. Oesophageal or gastric variceal bleeding identified on endoscopy or strongly suspected clinically;

3. Concomitant malignant disease of the upper gastrointestinal tract (e.g. gastric cancer);

4. History of major upper gastrointestinal surgery that substantially altered upper GI anatomy;

5. Pregnancy or lactation at the time of admission;

6. Self-discharge against medical advice or early discharge before completion of the necessary diagnostic work-up;

7. >20% missingness in key predictor variables, precluding reliable use in model development.

The above inclusion and exclusion criteria were applied identically to patients in the 2020–2023 development period and the 2024 temporal validation period.

## 3. Sample size and statistical power considerations

This study adopted a pragmatic cohort-based design without a formal a priori sample size calculation. Instead, we planned to include all consecutive eligible cases within the predefined time window to maximise the information available for model development and temporal validation.

- Between 1 January 2020 and 31 August 2024, a total of 1,235 adult patients with suspected UGIB were initially screened from the EMR.

- After applying the exclusion criteria, 965 patients with endoscopy-confirmed ANVUGIB were retained and constituted the final analysis cohort.

- Among these, 88 patients (9.1%) experienced rebleeding within 7 days of admission.

The final prediction model contained five independent predictors (syncope, pulse rate, red cell distribution width [RDW], serum albumin [ALB], and active bowel sounds). Based on 88 rebleeding events and five model degrees of freedom, the events-per-parameter ratio was approximately 17.6, which lies within commonly recommended ranges for multivariable prediction modelling.

To further mitigate the risk of overfitting and enhance the robustness of parameter estimates, we adopted the following strategies:

- We initially specified a clinically reasonable set of candidate predictors and then applied least absolute shrinkage and selection operator (LASSO) regression with 10-fold cross-validation to select variables for the final model;

- Patients from 2020–2023 were randomly split in a 7:3 ratio into a training cohort and an internal validation cohort, and model discrimination, calibration and decision curve performance were evaluated in the internal validation cohort;

- Calibration was further assessed using 1,000 bootstrap resamples;

- An independent temporal validation cohort (patients admitted in 2024) was used to examine the model’s temporal generalisability.

Taken together, the available sample size and the final model complexity appear reasonably aligned with the study aims. We explicitly acknowledge in the main text that larger, multicentre, prospective studies will be required for external validation and potential model updating.

## 4. Patient flow diagram

The patient selection process is summarised in Supplementary Figure S3 (patient flow diagram).

In brief:

- We first identified 1,235 adult patients with suspected UGIB admitted between January 2020 and August 2024 from the EMR;

- Applying the exclusion criteria (no confirmatory endoscopy within 48 hours, variceal bleeding, upper gastrointestinal malignancy, prior major upper GI surgery, pregnancy or lactation, self-discharge, and >20% missingness in key variables), 270 patients were excluded;

- The remaining 965 patients who fulfilled all inclusion and exclusion criteria and had endoscopy-confirmed ANVUGIB comprised the final study population.

These 965 patients were then allocated to the development dataset and the temporal validation cohort according to admission time, as detailed below and illustrated in Supplementary Figure S3.

## 5. Cohort construction: training set, internal validation set, and temporal validation cohort

### 5.1 Development dataset (2020–2023) and random split

The development dataset included all eligible patients admitted between 1 January 2020 and 31 December 2023, totalling 818 individuals. This dataset was used for model development and internal validation.

Within this dataset, we performed a 7:3 random split using computer-generated random numbers:

- Training cohort: 70% of patients (n = 572), used for LASSO-based variable selection and estimation of the final multivariable logistic regression model coefficients;

- Internal validation cohort: 30% of patients (n = 246), used to evaluate model discrimination, calibration and decision curve performance in a dataset drawn from the same time window but not used for model fitting.

No additional stratification factors were applied during randomisation, ensuring that both cohorts reflected the overall case mix of the development period.

### 5.2 Temporal validation cohort (2024)

The temporal validation cohort included all consecutive patients meeting the same inclusion and exclusion criteria who were admitted between 1 January 2024 and 31 August 2024, yielding 147 individuals. This cohort was entirely independent of model development and variable selection.

We applied the final model coefficients derived from the training cohort directly to these 147 patients to compute predicted 7-day rebleeding probabilities. Model performance in this cohort was assessed in terms of discrimination (AUC), calibration, and decision curve analysis, thereby evaluating the model’s temporal generalisability and robustness to changes in clinical practice over time.

### 5.3 Rationale for using temporal external validation

We chose a time-based split between the development and validation cohorts for the following reasons:

1. Alignment with real-world use: In clinical practice, prediction models are typically developed using historical data and subsequently applied to future patients, during which diagnostic and therapeutic strategies, medication patterns and case mix may evolve;

2. “Stress testing” under real-world conditions: Applying the model to consecutive cases from a later time period allows us to observe its performance under moderate temporal dataset shift and assess whether discrimination and calibration remain acceptable;

3. Informing model lifecycle management: Evidence of performance deterioration in the temporal validation cohort would signal the need for structured performance monitoring, timely recalibration, or retraining as part of the model’s longer-term maintenance.

We provide a detailed comparison of baseline characteristics between the development dataset (training + internal validation, total n = 818) and the temporal validation cohort (n = 147). In the main Results and Discussion sections, we further describe similarities and differences between the two cohorts, discuss the model’s temporal performance, and comment on potential model drift.

Supplementary Figure S3. Patient flow diagram for cohort selection.

Supplementary Table S1. Baseline characteristics of the development and temporal validation cohorts

| Variables | Total (n = 965) | Development cohort (n = 818) | Temporal validation cohort (n = 147) | P value |
| --- | --- | --- | --- | --- |
| Male, n (%) | 746 (77.3) | 626 (76.5) | 120 (81.6) | 0.174 |
| Age, （years）, Mean ± SD | 57.6 ± 18.9 | 57.9 ± 18.9 | 56.0 ± 18.9 | 0.271 |
| Hematemesis, n (%) | 311 (32.2) | 245 (30) | 66 (44.9) | < 0.001 |
| Melena, n (%) | 792 (82.1) | 669 (81.8) | 123 (83.7) | 0.583 |
| Syncope, n (%) | 37 (3.8) | 30 (3.7) | 7 (4.8) | 0.525 |
| Peptic ulcer history, n (%) | 212 (22.0) | 175 (21.4) | 37 (25.2) | 0.309 |
| Prior GI bleeding,  n (%) | 181 (18.8) | 153 (18.7) | 28 (19) | 0.922 |
| NSAID use, n (%) | 243 (25.2) | 196 (24) | 47 (32) | 0.039 |
| Antiplatelet use,  n (%) | 178 (18.4) | 135 (16.5) | 43 (29.3) | < 0.001 |
| Anticoagulant use,  n (%) | 44 (4.6) | 37 (4.5) | 7 (4.8) | 0.898 |
| Hypertension, n (%) | 462 (47.9) | 393 (48) | 69 (46.9) | 0.805 |
| Diabetes mellitus,  n (%) | 195 (20.2) | 160 (19.6) | 35 (23.8) | 0.237 |
| Ischemic heart disease, n (%) | 194 (20.1) | 165 (20.2) | 29 (19.7) | 0.902 |
| Smoking, n (%) | 555 (57.5) | 506 (61.9) | 49 (33.3) | < 0.001 |
| Alcohol use, n (%) | 538 (55.8) | 491 (60) | 47 (32) | < 0.001 |
| Altered mental status, n (%) | 166 (17.2) | 129 (15.8) | 37 (25.2) | 0.005 |
| Active bowel sounds, n (%) | 305 (31.6) | 257 (31.4) | 48 (32.7) | 0.767 |
| Hepatic dysfunction, n (%) | 28 ( 2.9) | 26 (3.2) | 2 (1.4) | 0.294 |
| Chronic kidney disease, n (%) | 61 ( 6.3) | 55 (6.7) | 6 (4.1) | 0.226 |
| Pulse, Median (IQR) | 81.0 (73.0, 91.0) | 80.0 (72.0, 91.0) | 82.0 (75.0, 92.0) | 0.31 |
| SBP（mmHg）, Median (IQR) | 123.0 (111.0, 136.0) | 123.0 (110.0, 136.0) | 123.0 (113.0, 136.5) | 0.71 |
| WBC（×109/L）, Median (IQR) | 7.9 (5.9, 10.4) | 7.9 (5.9, 10.6) | 8.1 (5.6, 9.7) | 0.312 |
| NEUT（×109/L）, Median (IQR) | 5.7 (3.9, 7.9) | 5.7 (3.9, 8.0) | 5.8 (3.9, 7.5) | 0.506 |
| RBC（×1012/L）, Median (IQR) | 3.3 (2.6, 4.0) | 3.3 (2.6, 4.0) | 3.1 (2.6, 3.8) | 0.138 |
| HB（g/L）, Median (IQR) | 95.0 (72.0, 117.0) | 96.0 (72.0, 118.0) | 90.0 (74.5, 111.0) | 0.26 |
| HCT（%）, Median (IQR) | 29.3 (22.8, 35.7) | 29.6 (22.8, 36.1) | 28.2 (22.7, 33.3) | 0.078 |
| RDW（%）, Median (IQR) | 13.5 (12.8, 14.9) | 13.5 (12.8, 14.9) | 13.6 (12.8, 14.9) | 0.837 |
| PLT（×109/L）, Median (IQR) | 209.0 (172.0, 259.0) | 209.5 (174.0, 261.0) | 205.0 (160.0, 254.0) | 0.243 |
| PT（s）,  Median (IQR) | 12.0 (11.3, 12.7) | 12.0 (11.3, 12.7) | 12.1 (11.3, 12.9) | 0.298 |
| INR, Median (IQR) | 1.1 (1.0, 1.2) | 1.1 (1.0, 1.2) | 1.1 (1.0, 1.2) | 0.041 |
| FIB（g/L）, Median (IQR) | 3.4 (2.9, 3.9) | 3.4 (2.9, 3.9) | 3.3 (2.8, 4.0) | 0.302 |
| D-dimer（ug/ml）, Median (IQR) | 0.1 (0.1, 0.3) | 0.1 (0.1, 0.3) | 0.1 (0.0, 0.3) | 0.326 |
| CRP（mg/L）, Median (IQR) | 2.0 (0.5, 6.3) | 1.7 (0.5, 5.8) | 2.9 (0.9, 7.9) | 0.027 |
| ALT（U/L）, Median (IQR) | 16.0 (11.0, 23.0) | 16.0 (11.0, 23.0) | 16.0 (11.8, 22.0) | 0.754 |
| AST（U/L）, Median (IQR) | 20.0 (17.0, 25.0) | 20.0 (17.0, 26.0) | 20.0 (17.0, 24.5) | 0.867 |
| ALB（g/L）, Median (IQR) | 38.5 (34.7, 42.1) | 38.5 (34.7, 42.5) | 38.4 (34.6, 40.3) | 0.068 |
| BUN（mmol/L）, Median (IQR) | 9.3 (6.3, 13.7) | 9.5 (6.3, 13.8) | 8.9 (6.7, 13.0) | 0.483 |
| Cr（umol/L）, Median (IQR) | 72.0 (59.0, 90.0) | 72.0 (59.0, 91.0) | 69.0 (62.0, 80.0) | 0.056 |
| Ca2+（mmol/L）, Median (IQR) | 2.1 (2.1, 2.3) | 2.2 (2.1, 2.3) | 2.1 (2.0, 2.2) | < 0.001 |
| K+（mmol/L）, Median (IQR) | 4.1 (3.8, 4.4) | 4.1 (3.8, 4.4) | 4.0 (3.7, 4.4) | 0.306 |
| GBS, Median (IQR) | 8.0 (6.0, 11.0) | 8.0 (6.0, 11.0) | 9.0 (6.0, 10.5) | 0.928 |
| AIMS65, Median (IQR) | 1.0 (0.0, 2.0) | 0.0 (0.0, 1.0) | 1.0 (0.0, 2.0) | < 0.01 |
| Rebleeding, n (%) | 88 (9.1) | 75 (9.2) | 13 (8.8) | 0.9 |

Values are presented as mean ± standard deviation (SD), median (interquartile range, IQR), or number (percentage), as appropriate. Continuous variables were compared using the t-test or Mann–Whitney U test, and categorical variables were compared using the χ² test or Fisher’s exact test.
